# Supplementary material for: Immune Checkpoint Inhibitors in Oral Cavity Squamous Cell Carcinoma and Oral Potentially Malignant Disorders: A Systematic Review
Source: Cancers (Basel). 2020 Jul 17;12(7):1937. doi: 10.3390/cancers12071937 (PMC7409293; doi:10.3390/cancers12071937)
Supplement: Supplementary file 1 [file cancers-12-01937-s001.pdf]

Table S1. Summary of inclusion criteria data from the included articles.

#	Author/Year	Average age (yr)	Gender (M/F)	Sampl e size	OSCC or OPMD	Follow-up period	Specimen type	Biomarker	Detection method	Control
1	Aggarwal et al. 2017 [1]	Not Specified	Not Specified	53	OSCC	Not Specified	Peripheral blood	CTLA4	Immunoblotting	Age and sex-matched healthy tissue samples
2	Ahn et al. 2017 [2]	57.7 (23–84)	45/23	68	OSCC	44.3 m	FFPE	PD-L1	PCR + IHC	Chorionic villi of human placenta
3	Balermpas et al. 2017 [3]	57	71/17	161	OSCC	48 m	FFPE	PD-1 + PD-L1	IHC	-
4	Bauml et al. 2017 [4]	61 (33–90)	138/33	171	OSCC	Not Specified	Not Specified	PD-L1	Not Specified	Not Specified
5	Bharti et al. 2013 [5]	50.6 (20–88)	117/13	130	OSCC	Not Specified	Venous blood	CTLA4	PCR-RFLP	Normal Tissue
6	Bhosale et al. 2017 [6]	49 (40–59)	299/93	481	OPMD	Not Specified	FFPE and Frozen	CD274 (PD-L1)	RT-qPCR	Not Specified
7	Cai et al. 2019 [7]	Not Specified	28/12	40	OSCC	Not Specified	FFPE	PD-L1	IHC	Normal mucosal tissues
8	Chen et al. 2015 [8]	51 (26–81)	190/28	218	OSCC	31 m	FFPE	PD-L1	IHC	Not Specified
9	Chen et al. 2018 [9]	58.8	99/7	106	OSCC	Not Specified	FFPE	PD-L1	IHC	Not Specified
10	Chen et al. 2019 [10]	55 (24–79)	52/35	62	OSCC and OPMD	Not Specified	FFPE	PD-L1	IHC	Normal tissue
11	Cho et al. 2011 [11]	59	32/13	45	OSCC	Not Specified	FFPE	PD-L1	IHC	Skeletal muscle tissue
12	Dong et al. 2017 [12]	60 (45–72)	15/5	20	OSCC	Not Specified	Peripheral blood	Tim-3	Cell sorting	Normal tissue
13	Du et al. 2011 [13]	44.8 (23–72)	36/24	60	OPMD	Not Specified	FFPE + Bloods	PD-L1	IHC, RT-PCR	Normal tonsillar tissue

14	Falco et al. 2019 [14]	63 (36–84)	28/12	40	OSCC	6 m	Not Specified	PD-L1	Not Specified	Not Specified
15	Fayette et al. 2017 [15]	Not Specified	24/7	31	OSCC	442 days	Not Specified	Not Specified	Not Specified	Not Specified
16	Feldman et al. 2015 [16]	59.5 (19–90)	570/165	735	OSCC	Not Specified	FFPE	PD-1, PD-L1	IHC	protein specific controls
17	Ferris et al. 2017 [17]	60 (28–83)	300/61	361	OSCC	5.1 m	Fresh or archived	PD-L1	IHC	Tumours treated with standard therapy (methotrexate, docetaxel, cetuximab)
18	Fiedler et al. 2017 [18]	60.5 (43.3–83.6)	73/9	82	OSCC	Not Specified	FFPE	PD-1, PD-L1	IHC + cell counting. High expression was classified as a minimum of 5% positivity among counted cells	Not Specified
19	Foy et al. 2017 [19]	Not Specified	Not Specified	212	OSCC	Not Specified	FFPE	PD-L1	IHC	Three independent cohorts were used as validation cohorts
20	Gasparoto et al. 2010 [20]	58.42 (41–96)	Not Specified	12	OSCC	Not Specified	Fresh	CD152 (CTLA4)	Flow cytometry	Aged matched healthy gingival tissue
21	Ghapanchi et al. 2019 [21]	39 (27–105)	14/90	104	OPMD	Not Specified	Bloods	PD-1	Genotyping	Normal tissue
22	Goltz et al. 2017 [22]	61.9 (59.1–62.8)	94/26	120	OSCC	32 (29-34) m	FFPE	PD-1	RT-PCR	validation cohort
23	Groeger et al. 2016 [23]	61.5 (40–79)	12/3	15	OSCC	Not Specified	FFPE, frozen	B7-H1 (PD-L1)	IHC	Non-tumour areas of sampled tissue
24	Hanna et al. 2017 [24]	36 (15–45)	49/32	81	OSCC	74 m	FFPE	PD-L1	IHC, Whole exome sequencing + genomic analysis	Not Specified
25	Hanna et al. 2017 [25]	57.5 (26–84)	23/11	34	OSCC	9 m	Fresh	PD-1, Tim3	Flow cytometry	Isotype controls

26	Jie et al. 2013 [26]	64.7 (40–83)	15/10	27	OSCC	Not Specified	Peripheral venous blood	PD-1, CTLA-4, Tim-3, Lag-3	Flow cytometry	Isotype controls
27	Kämmerer et al. 2010 [27]	63.8 (44–86)	59/24	83	OSCC	Not Specified	PBMCs	CTLA4	RT-PCR	Normal tissue
28	Katou et al. 2007 [28]	Not Specified	Not Specified	29	OPMD	Not Specified	Frozen	PD-1	IHC + PCR	Normal lingual mucosa
29	Larkins et al. 2017 [29]	60 (20–84)	139/35	192	OSCC	Not Specified	Not Specified	PD-1	Not Specified	Not Specified
30	Lecerf et al. 2019 [30]	56	77/19	96	OSCC	Not Specified	FFPE	PD-1, PD-L1, CTLA-4	IHC, RT-qPCR	Not Specified
31	Lechner et al. 2017 [31]	68 (49–85)	27/7	34	OSCC	Not Specified	FFPE, Peripheral blood	PD-1, PD-L1, CTLA4	Flow cytometry, IHC and PCR	Normal tissue
32	Leduc et al. 2017 [32]	54 (37–84)	14/7	21	OSCC	Not Specified	FFPE	PD-L1	IHC	Not Specified
33	Lin et al. 2015 [33]	56	236/69	305	OSCC	3.8 years	FFPE	PD-L1	IHC	Normal tonsil
34	Linedale et al. 2017 [34]	70.2	14/2	17	OSCC	Not Specified	FFPE, Peripheral blood	PD-1, PD-L1, Tim3 and CTLA4	Flow Cytometry and IHC	Normal tissue
35	Malaspina et al. 2011 [35]	58.42 (41–96)	46/13	61	OSCC and OPMD	Not Specified	Frozen tissue + Bloods	PD-1 + PD-L1	IHC, Flow cytometry	Age matched normal tissue
36	Malm et al. 2015 [36]	Not Specified	Not Specified	451	OSCC	Not Specified	Peripheral blood	PD-1 , PD-L1 + Lag-3	IHC, Flow cytometry	Isotype controls
37	Maruse et al. 2018 [37]	64.0 (19–88)	70/27	97	OSCC	Not Specified	FFPE	PD-1, PD-L1	IHC	Staining with phosphate buffered saline only
38	Mattox et al. 2017 [38]	Not Specified	Not Specified	53	OSCC	Not Specified	FFPE	PD-L1	IHC-IF	Isotype controls
39	Moratin et al. 2019 [39]	64.3 (27–88)	105/70	175	OSCC	Not Specified	FFPE	PD-L1	IHC	Not Specified
40	Moreira et al. 2010 [40]	60	18/4	26	OSCC	Not Specified	FFPE	CTLA4	IHC	Omission of primary antibodies

41	Muller et al. 2017 [41]	1st cohort 64.27 (38–88). 2nd cohort 62.41 (27–87)	82/16 142/53	293	OSCC	1st cohort 573.93 days. 2nd cohort 856 days	FFPE	PD-L1	IHC	Not Specified
42	Naruse et al. 2019 [42]	64	65/56	121	OSCC	Not Specified	FFPE	PD-1, PD-L1	IHC	Normal tissue
43	Ngamphaiboon et al. 2019 [43]	65 (28–89)	145/58	203	OSCC	Not Specified	FFPE	PD-L1	IHC	Not Specified
44	Okada et al. 2018 [44]	65 (32–80)	23/3	26	OSCC	47 m	FFPE	PD-L1	IHC	Not Specified
45	Oliveira-Costa et al. 2015 [45]	61.8 (34–95)	92/100	180	OSCC	20 m	Frozen	PD-L1	RT-qPCR, IHC	Normal oral epithelium
46	Pekiner et al. 2012 [46]	51.1	9/21	30	OPMD	Not Specified	Peripheral blood	CTLA4	Flow cytometry	Normal tissue
47	Poropatich et al. 2017 [47]	61.1	19/7	30	OSCC	Not Specified	Frozen	PD-1, CTLA4 and TIM3	Flow cytometry	Normal tissue + Tonsil
48	Quan et al. 2016 [48]	49.1	17/5	22	OSCC	Not Specified	FFPE, Frozen, PBMC	Tim3, PD-1	IHC, Flow cytometry	Matched PBMCs
49	Rasmussen et al. 2019 [49]	Not Specified	16/12	28	OSCC	Not Specified	FFPE	PD-L1	IHC	Not Specified
50	Ryu et al. 2017 [50]	58	296/97	393	OSCC	Not Specified	FFPE	PD-1	IHC	Not Specified
51	Saâda-Bouzid et al. 2017 [51]	63	27/7	34	OSCC	10.3 m	Not Specified	PD-1 PD-L1	Not Specified	Not Specified
52	Sablin et al. 2016 [52]	56	77/19	96	OSCC	125 m (2.6d – 236 m)	Frozen	PD-L1	IHC	Adjacent healthy tissue
53	Satgunaseelan et al. 2017 [53]	65 (30–89)	130/87	217	OSCC	22 m (1 m-12yrs)	FFPE	PD-L1	IHC	Not Specified
54	Scognamiglio et al. 2017 [54]	Not Specified	Not Specified	96	OSCC	Not Specified	FFPE	PD-L1	IHC	Normal tonsil tissue

55	Seiwert et al. 2016 [55]	63 (20–83)	49/11	60	OSCC	14 m	Not Specified	PD-L1	IHC	Not Specified
56	Shayan et al. 2017 [56]	61 (43–75)	9/5	14	OSCC	Not Specified	Peripheral blood	PD-L1, CTLA-4	Flow cytometry	Isotype control
57	Singh et al. 2017 [57]	52.25 (40–68)	7/5	12	OSCC	Not Specified	Fresh	PD-L1	qPCR	Adjacent normal tissue
58	Sridharan et al. 2016 [58]	59	18/2	20	OSCC	14 m	Peripheral blood	PD-1, Tim3, PD-L1	Flow cytometry, cytokine assay	Normal tissue
59	Strati et al. 2017 [59]	65	75/19	113	OSCC	18.9 m	Peripheral blood	PD-L1	RT-qPCR	Normal tissue
60	Straub et al. 2016 [60]	57 (38–86)	54/26	80	OSCC	31 m (2-63 m)	FFPE	PD-1, PD-L1	IHC, ISH	Normal tissue
61	Strauss et al. 2017 [61]	58 (36–75)	6/4	10	OSCC	Not Specified	Peripheral venous blood	CTLA-4	Flow cytometry	Aged matched normal tissue
62	Takahashi et al. 2019 [62]	69 (33–92)	50/27	77	OSCC	Not Specified	FFPE	PD-L1	IHC	Not Specified
63	Takakura et al. 2017 [63]	nNAC 65 (32-81). NAC = 71 (54-85)	NAC = 3/5, nNAC = 4/6	18	OSCC	Not Specified	FFPE	PD-1 + PD-L1	IHC	Not Specified
64	Troeltzsch et al. 2017 [64]	61.34	48/40	88	OSCC	Not Specified	FFPE	PD-L1	IHC	Tonsillar tissue
65	Wang et al. 2019 [65]	67	20/16	36	OSCC	Not Specified	FFPE	PD-L1, TIM3, CTLA4, Lag-3	IHC	Not Specified
66	Weber er al. 2018 [66]	64.6 (35–93)	29/16	45	OSCC	Not Specified	Fresh, Peripheral blood	PD-L1	RT-qPCR	healthy controls
67	Wirsing et al. 2018 [67]	59	43/32	75	OSCC	Not Specified	FFPE	PD-L1	IHC, RT-qPCR	Not Specified
68	Wong et al. 2006 [68]	51.4 (30–75)	110/8	118	OSCC	3 years	Fresh	CTLA4	PCR	Patients without OSCC

69	Wu et al. 2017 [69]	Not Specified	Not Specified	549	OSCC	Not Specified	FFPE	PD-L1 and CTLA4	IHC	43 normal oral mucosae
70	Wu et al. 2019 [70]	Not Specified	Not Specified	16	OSCC	Not Specified	Peripheral blood	PD-1	Flow cytometry	Normal oral mucosa
71	Xu et al. 2019 [71]	Not Specified	Not Specified	53	OSCC	Not Specified	FFPE	PD-1, PD-L1	IHC	Not Specified
72	Yagyuu et al. 2017 [72]	62.6 (8-86)	42/78	120	OPMD	45.6 m	Fresh	PD-L1	IHC	Tonsil
73	Yang et al. 2019 [73]	Not Specified	Not Specified	279	OSCC	Not Specified	FFPE	PD-1, Tim3, Lag3	IHC	42 normal mucosa
74	Yoo et al. 2019 [74]	59 (20–89)	114/44	158	OSCC	54.5 m	FFPE	PD-L1	IHC	Isotype controls
75	Youngnak-Piboonratanakit et al. 2004 [75]	62.6 (27–64)	42/78	13	OPMD	45.6 m	Fresh	PD-1	IHC	Isotype controls, normal tissue
76	Zhou et al. 2012 [76]	42 (22–65)	10/12	22	OPMD	Not Specified	Peripheral blood	PD-1 and PD-L1	Flow cytometry, ELISA	healthy patients

Abbreviations: oral cavity squamous cell carcinoma (OSCC), fixed formalin paraffin imbedded (FFPE), immunohistochemistry (IHC), oral leukoplakia (OLK), polymerase chain reaction (PCR), real time quantitative PCR (RT-qPCR) restriction fragment length polymorphism (RFLP), in situ hybridization (ISH), immunofluorescence (IF), tumour infiltrating lymphocyte (TIL), peripheral blood mononuclear cell (PBMC), neoadjuvant chemotherapy (NAC), non-neoadjuvant chemotherapy (nNAC).

Table 2. Summarized results of included articles.

#	Author/Year	Pattern of expression	Main findings
1	Aggarwal et al. 2017 [1]	CTLA-4 Tregs exhibited a higher prevalence in the peripheral circulation compared to normal controls	CTLA-4 is an important functional marker of Tregs.
2	Ahn et al. 2017 [2]	High PD-L1 expression was a favourable prognostic factor for overall survival only in the miR-197 high subgroup	PD-L1 expression on IHC is associated with increased TILs and favourable prognosis in miR-197 high subgroup
3	Balermpas et al. 2017 [3]	PD-1 expression was defined as being low in 88 patients and high in 73 patients	PD-L1 in CD8 cells represents a promising prognostic marker and could be used to guide treatment with PD-1/PD-L1 inhibitors
4	Bauml et al. 2017 [4]	17% of patients with PD-L1 expression of >1% of tumour cells responded to treatment with nivolumab	pembrolizumab exhibited clinically significant antitumor activity and an acceptable safety profile in heavily pretreated OSCC
5	Bharti et al. 2013 [5]	High frequency of 1661G allele and AG genotype may be related to increased risk while low frequency of A allele suggests it to be protective in patients with tobacco related OSCC	Association between several polymorphisms in CTLA-4 gene and OSCC supports the role of immune/inflammatory molecules in the susceptibility to tobacco-related OSCC
6	Bhosale et al. 2017 [6]	CD274 expression was amplified in OSCC compared to normal tissue. CD274 was dysregulated in OSCC compared to leukoplakia	CD274 and its ligand PD-1 are important targets of immunotherapy in OSCC
7	Cai et al. 2019 [7]	PD-L1 was positive in 58% of tumour samples, compared to 0% in normal mucosa. In the tumour samples, PD-L1 was found to localize to cell membrane and cytoplasm and was highly expressed in poorly differentiated tumours. However, PD-L1 was weakly expressed in well and moderately differentiated tumours	anti-PD-1 mAb may have better tumour permeability due to its smaller molecular weight. It may be more efficacious in poorly differentiated OSCCs with PD-L1 higher expression.
8	Chen et al. 2015 [8]	Patients with both necrosis and positive PD-L1 expression in OSCC surrounding necrosis had worse outcome and disease control	The aggressive behaviour of advanced OSCC could be related to PD-L1 immune escape, therefore patients with positive tumour PD-L1 expression may be good candidates for anti-PD-L1 immunotherapy.
9	Chen et al. 2018 [9]	72 samples were PD-L1 positive and 34 were PD-L1 negative. Positive p16INK4A expression was significantly higher and the mean age of patients was significantly higher in the group exhibiting positive expression of PD-L1 compared with the negative group.	This study identified an association between PD-L1 and p16INK4A expression in non-OPHNSCC

10	Chen et al. 2019 [10]	PD-L1 was significantly associated with the OSCC pathological grade, and higher PD-L1 staining was observed in OSCC compared to OLK	PD-L1 expression in OSCC and OLK was closely associated with disease progress and CD8+ TILs
11	Cho et al. 2011 [11]	PD-L1 expression on OSCC cells was observed in 87% of cases. The staining patterns were membranous and/or cytoplasmic. The density of intratumoral CD8+ T lymphocytes showed a significant inverse correlation with the PD-L1 expression of tumour cells	The association between PD-L1 and CD8 T cells+ may be relevant to the PD-L1/PD-1 interactions resulting in the negative regulation of activated T-cells. PD-L1 expression was also correlated with the histological grade of the tumours
12	Dong et al. 2017 [12]	Tim-3+ monocytes suppressed IFN-γ secretion in CD8+ T cells	Blocking Tim-3 and/or Gal-9 could inhibit the Tim-3/Gal-9-mediated suppression of monocytes in vitro
13	Du et al. 2011 [13]	PD-L1 was abundantly expressed on keratinocytes and slightly on the infiltrate T cells in the subepithelium	PD-L1 was abundantly expressed in most OLP cases. PD-L1 mRNA levels in OLP patients' mucosa and blood were 1.43- and 0.62-fold compared to the control
14	Falco et al. 2019 [14]	Not Specified	22 patients achieved clinical benefit. Partial response seen in 10, stable disease 9, Complete response in 3 patients. For those with high PD-L1 expression, single-agent pembrolizumab also improves overall survival compared with Cetuximab plus chemotherapy.
15	Fayette et al. 2017 [15]	Not Specified	Cabazitaxel gave a signal of activity OSCC but was toxic. In future studies, Cabazitaxel could be used at 20 mg/m2 every 3 weeks or weekly at a lower dose like Paclitaxel or Docetaxel.
16	Feldman et al. 2015 [16]	PD-1: 125/182 altered expression. PD-L1: 32/183 altered expression. PD-1-positive TILs were detected in a range of 65% to 72%, across disease stages. Both PD-1-positive TILs and PD-L1-positivity in tumour cells was found across primary disease sites	The data supports the use of agents in clinical trials (PIK3CA, PD-1/PDL1), combination strategies (PIK3CA1/EGFR), or agents approved for other solid tumours (MGMT, HER2). We propose a comprehensive molecular profiling approach to enhance personalized therapy options for OSCC.
17	Ferris et al. 2017 [17]	Tumour PD-L1 expression status could be evaluated in 260 of 361 patients (72.0%). 57.3% of evaluated patients had a tumour membrane PD-L1 expression level of 1% of cells per field of view (minimum 100 cells) or more.	Exploratory biomarker analysis indicated that patients who were treated with nivolumab had an average of 4.83 months longer overall survival than those treated with standard therapy, regardless of tumour PD-L1 expression, and nivolumab-treated patients had a risk of death that was 30%

			lower than the risk among patients assigned to standard therapy
18	Fiedler et al. 2017 [18]	43 Low 39 high PD-1 expression. 50 low 31 high PD-L1 expression. 33 low 48 high PD-L1 TI positive TILs. 39 samples showed high PD-1 expression. High PD-L1 expression of tumour cells was found in 31 samples and 48 samples showed a high expression of PD-L1+ TILs	Results show a nearly significant association between PD-L1 expression in tumour cells and complete tumour response upon irradiation (77.4% high PD-L1 showed complete response vs 54.0% of low PD-L1 expression). PD-L1 was expressed in 40% of patients indicating radiosensitivity and favourable survival in this group of patients
19	Foy et al. 2017 [19]	PD-L1 was overexpressed in never smokers/drinkers compared to smokers/drinkers and in never smokers/drinkers at protein level. PD-L1-positive immune cells were mostly adjacent to tumour cells expressing PD-L1.	OSCC in never smokers/drinkers are characterized by an enrichment of PD-1 pathways, a higher intratumor T-cell infiltrate, an overexpression of IDO1 and PD-L1, and higher score of response signature to pembrolizumab
20	Gasparoto et al. 2010 [20]	39.3 ± 7.1% of gated CD4+CD25+ T cells expressed CTLA-4.	The results confirm that CD4+CD25+ T cells, but not CD4+CD25- T cells, from OSCC patients have potent inhibitory consequences on T effector function and promote the generation of IL-10
21	Ghapanchi et al. 2019 [21]	At position PD-1.3, the genotype of GG was present in 80.8%, while the genotypes of GA and AA were found in 13.7% and 5.5% patients. The most prevalent genotype among patients was CT heterozygote.	Polymorphism of PD-1.3 and PD-1.5 genes did not have any significant correlation with OLP susceptibility
22	Goltz et al. 2017 [22]	80 patients had high mPD-1 expression, 40 had low mPD-1 expression. mPDCD1high was associated with shorter overall survival	mPDCD1 might potentially serve as a predictive biomarker for the response to immunotherapies targeting the PD-1/PD-L1 axis
23	Groeger et al. 2016 [23]	All 15 oral squamous cell carcinomas investigated showed a positive expression of the PD-L1 receptor in the cancerous areas	The 5-year survival rate of the patients whose tissues were positive for PD-L1 expression, was 73.33 % (11 of 15). Expression of PD-L1 may be a prognostic marker in oral squamous cell carcinomas.
24	Hanna et al. 2017 [24]	Immunohistochemistry for tumoral PD-L1 in a subset of female patients revealed that 86.9% had >10% (range 0–100%) expression on tumour cells, with higher rates of membranous vs. cytoplasmic staining. PD-L1 expression on TILs was negligible	Female OSCC patients with greater membranous PD-L1 positivity and the presence of TILs showed a decreased risk of recurrence and improved survival, hazard ratio 0.58, p<0.001
25	Hanna et al. 2017 [25]	A subgroup of tumours with an inflamed immune composition characterized by a robust CD8+ T cell infiltrate with high checkpoint co-expression (PD-1/TIM3+) independent of HPV or	Recurrent/metastatic OSCC patients with an inflamed immunophenotype treated with single agent PD-1 blockade appeared to benefit

		smoking status. Recurrent/metastatic OSCC demonstrated improved survival in those patients with tumours exhibiting >1% PD-L1 expression	
26	Jie et al. 2013 [26]	CTLA-4 and CD39 are co-expressed on the majority of intratumoral FOXP3+ Tregs	The frequency of cells expressing CTLA-4, TIM-3 and PD-1 is significantly increased on intratumoral Tregs compared with circulating Tregs
27	Kämmerer et al. 2010 [27]	A significant difference was found for CTLA-4 1661 A/G genotype between the patients and the controls (30.1% vs 10.0%) but not for CTLA-4 +49 A/G.	Potential for treating OSCC patients by targeting the CTLA-4 –1661 A/G genotype, expressed in approximately 3x more tumour samples than in controls.
28	Katou et al. 2007 [28]	Intraepithelial CD8+ TILs express PD-1 at a high rate, and the stromal CD8+ TILs, as well as CD8+ T cells in OLP, express PD-1 at low rates. Expression of PD-1 in OLP ~100x10^-5, tongue cancer ~500x10^-5, compared to normal ~10x10^-5.	High expression rate of PD-1 by the intraepithelial CD8+ TILs suggests their exhausted functions
29	Larkins et al. 2017 [29]	Not Specified	Accelerated approval expands the FDA-approved indications for pembrolizumab for the treatment of recurrent or metastatic OSCC with disease progression on or after platinum-containing chemotherapy
30	Lecerf et al. 2019 [30]	85 tumours overexpressed at least one of TIGIT, CTLA4, PD-1/PD-L1 and OX40/OX40L and 33 tumours simultaneously overexpressed them all. No tumour exclusively overexpressed PD-1 or OX40. PD-L1 was exclusively overexpressed in only one sample	PD-1 overexpression was associated with good prognosis, whereas low mRNA levels of PD-1 correlated with poor prognosis and high risk of recurrence
31	Lechner et al. 2017 [31]	Significantly higher rate of PD-1 and CTLA-4 expression on TILs. The percentage of PD-1, PD-L1 and CTLA-4 expressing circulating T cells was increased in OSCC patients compared to healthy donors, indicating elevated proportions of regulatory or exhausted T-cell phenotypes	Results demonstrate elevated proportions of regulatory or exhausted T-cell phenotypes in OSCC patients compared to healthy controls, with 3.7% of total cells compared to 1.6%, respectively
32	Leduc et al. 2017 [32]	A significant increase in PD-L1 expression was observed in both tumour and immune cells post-TPF induction chemotherapy, with increases from 9.5% positivity prior to treatment compared to 38% positivity at the 5% cut-off level	Results suggest combination strategies of concomitant administration of cytotoxic therapies and anti-PD-1/PD-L1 therapies might be relevant for OSCC
33	Lin et al. 2015 [33]	High PD-L1 cytoplasm intensity was more likely in tumours from female than from male patients. High expression levels of PD-L1 were also more likely to occur in tumours from female	High PD-L1-expression was significantly associated with distant metastasis and poor prognosis in male patients and smoking patients Results suggest patients with high PD-L1

		than from male patients. High PD-L1 expression is an independent risk factor in males (hazard ratio = 1.556) and smokers (hazard ratio = 2.058)	expression had poor clinical outcome (hazard ratio = 2.74) and might require PD-L1-targeted immunotherapy to improve their prognosis
34	Linedale et al. 2017 [34]	Amongst the CD8 infiltrate, the frequency of PD-1, CTLA-4 and Tim-3 expressing cells was significantly elevated in tumour relative to the blood across the patient	Results suggests an enrichment of PD-1+ CD8 T cells in perineural tumour tissue relative to blood and that Tim-3 expressing T cells are enriched in perineural tumour and might be good candidates for targeted antibody therapy
35	Malaspina et al. 2011 [35]	Numbers of CD8 T cells expressing PD-1 were significantly higher in OSCC patients than in control subjects. OSCC tumour samples contained elevated expression of PD-1 and present higher number of CD4+ PD-1+ T cells when compared with tissue from actinic chelitis patients	PD-1 and PD-L1 molecules are present in different phenotypes of lymphocytes in blood actinic chelitis and OSCC. High PD-1 expression in CD4+ (43%) and CD8+ (68%) T cells may be used as a potential prognostic marker in oral tumours or in pre-malignant lesions
36	Malm et al. 2015 [36]	Abundant PD-1 expression on CD4 and CD8 T cells at all sites, significantly higher than Lag-3. PD-1 was expressed at some level on both CD4 and CD8 T cells in all samples.<br>11% of analysed tumours were PD-L1 negative, 56% had regional expression, and 44% had diffuse expression with infiltrating lymphocytes	PD-1 is expressed on T cells from HPV-negative patients with OSCC and the abundance of its ligand in tumour tissue between 44-56%.
37	Maruse et al. 2018 [37]	PD-L1 and PD-1 expression was not observed in the adjacent non-malignant oral epithelium. Increased expression of PD-L1 and PD-1 was significantly associated with 3.67 times greater incidence of cervical lymph node metastasis	Patients with PD-L1-positive expression had a significantly more unfavourable outcome than those with PD-L1-negative expression (69.2% vs 91.0% 5 year survival). The co-expression of PD-L1 and PD-1 is predictive of a poor prognosis in OSCC patients. PD-L1 expression in cancer cells is more critical than PD-1 expression in the infiltrating inflammatory cells in the prediction of the prognosis of OSCC patients.
38	Mattox et al. 2017 [38]	42/42 samples expressed PD-L1 at >1% of membranous expression by tumour and/or immune cells. PD-L1 and CD4 and CD8 TIL expressions were not significantly associated with clinical outcomes	Our study suggests that tumour-specific CD4+ T cells may be a key regulator of PD-L1 expression within the Tumour microenvironment.
39	Moratin et al. 2019 [39]	Expression levels of PD-L1 were significantly higher in female patients than male patients (9% compared to 7.2%). Mean expression of PD-L1 was 7.9% of cells per section	The expression scores of PD-L1 correlated significantly with the tumour size, the presence and the stadium of neck node metastases, clinical stage, and age. There was a trend

			toward worse overall survival for patients with higher PD-L1 expression<br>AntiPD-1/PD-L1 therapy may be of therapeutic use even in early stage OSCC to prevent further disease progression
40	Moreira et al. 2010 [40]	A lower percentage of CTLA-4+ cells was observed in OSCC compared with Lip SCC (3.39% vs 13.12%).	Higher CTLA-4 expression was significantly associated with a low tumour proliferative index<br>No association between CTLA-4 expression and survival
41	Muller et al. 2017 [41]	PD-L1 expression was at the membrane and cytoplasm of tumour cells. A subset of peritumoral and tumour infiltrating lymphocytes also revealed strong immunoreactivity for PD-L1.	There was no correlation between PD-L1 with tumour stage, lymph node involvement, lymphatic invasion, vascular invasion, tumour grade or extracapsular expansion<br>PD-L1 expression is a suitable prognostic biomarker, independently of other well-known prognostic factors such as tumour stage and tumour grade (hazard ratio: 4.269 and 2.845 for cohort 1 and 2). PD-L1 expression was a strong predictor for poor outcome
42	Naruse et al. 2019 [42]	Among 121 OSCC, 54.5% were positive for PD-1 and 57.9% were positive for PD-L1 by IHC.<br>PD-L1 was expressed primarily in the cytoplasm and nuclei of tumour cells, with particularly strong expression observed at the invasive front.	A significant decrease in 5-year disease specific survival rate for patients with combined PD-1+/PD-L1+ expressions.<br>Inhibition of the PD-1/PD-L1 axis may be a good strategy to improve prognosis in OTSCC patients with local recurrence after NAC
43	Ngamphaiboon et al. 2019 [43]	79 of 94 OSCC samples (84%) were classed as positive for PD-L1 expression.	Patient sex, smoking status, site of primary cancer, stage at diagnosis, and p16 status were not associated with PD-L1 expression<br>Highly expressed PD-L1 (≥50%) was an independent prognostic factor for poor overall survival in anti-PD-1/PD-L1 untreated OSCC patients
44	Okada et al. 2018 [44]	PD-L1 expression was negative in 11 cases, weakly positive in 9 cases, and strongly positive in 6 cases.	The 5-year overall survival rates in the low and high PD-L1 groups were 72.5% and 16.7%, respectively.<br>High PD- L1 expression is an independent poor prognostic factor in OSCC patients with surgically resected pulmonary metastasis
45	Oliveira-Costa et al. 2015 [45]	Cytoplasmic PD-L1 expression in 47/96 cases while membrane expression was in 7/96. PD-L1 cytoplasmic expression was histologically found in areas with undifferentiated cells	Cytoplasmic PD-L1 expression was significantly associated with tumour size (hazard ratio: 7.618). PD-L1 was expressed

			translationally and at the protein level in OSCC-derived circulating tumour cells.
46	Pekiner et al. 2012 [46]	CTLA-4 was expressed an average of 1.01 times greater than CD8 in OLP, but an average of 0.74 times lower in controls	No significant difference in the percentage of CTLA-4 in OLP and controls (P>0.05)
47	Poropatich et al. 2017 [47]	PD-1+ CD8+ T-cell levels were positively correlated with increased size of the primary site tumour (r = 0.63). CTLA-4 and PD-1 expression on CD8+ T cells were significantly higher in stage IV HPV-negative than in stage IV HPV-positive patients. Treg TIM3 expression was 51.94% in HPV-negative compared to 14.88% in HPV-positive samples	PD-1 and TIM-3 T-cell expression were specifically elevated in OSCC patients as compared with healthy controls
48	Quan et al. 2016 [48]	Compared with the matched PBMCs in both CD41 and CD81 TIL-Ts we found a significant increase in the expression of PD-1 and Tim-3	T-cells expressing PD-1 and Tim-3 indicate T cell exhaustion and contribute to an immunosuppressive environment
49	Rasmussen et al. 2019 [49]	Using a 1% cut-off value to define positivity, 36% of the specimens were concordant with tumour proportion score and 52% were concordant with combined positive score. With a 50% cut off, the concordance was higher at 70% with tumour proportion score and 54% with combined positive score.	The assessed PD-L1 positivity varies markedly within the tumour in this patient series which limits the utility of this biomarker
50	Ryu et al. 2017 [50]	When comparing immune cell compositions among p16 patterns, both the MOSAIC and STRONG patterns similarly revealed high infiltration of CD3+, CD8+, and PD-1+ T cells. PD-1+ T cells were higher in NUCLEAR than in the ABSENT pattern s of staining	The association of the nuclear pattern with worse prognosis may be related to a relatively high proportion of exhausted and dysfunctional PD-1+ T cells, although with fewer infiltrating CD8+ cytotoxic T cells
51	Saâda-Bouzid et al. 2017 [51]	Not Specified	29% of patients with OSCC experienced hyperprogression as defined by a tumour growth kinetics ratio exceeding two during anti-PD-1/PD-L1 therapy
52	Sablin et al. 2016 [52]	27 samples showed PD-L1 overexpression and 69 samples showed no PD-L1 overexpression	Identification of druggable overexpressed genes PGF, PDL1, CDK6, EGFR, MET, VEGFA were associated with a poor outcome.
53	Satgunaseelan et al. 2017 [53]	PD-L1 expression in more than 5% of the SCC cells was present in 40 cases. Cell membrane and cytoplasmic staining were observed in this cohort and there was no nuclear staining. Weak 1+ staining intensity was observed in 26, moderate 2+ staining	PD-L1 expression was observed in 18% of the cases in the current cohort

		intensity was present in 7 and strong 3+ staining intensity was seen in 7 cases	
54	Scognamiglio et al. 2017 [54]	Fifty-eight of 96 cases (60%) showed PD-L1 expression in the immunocytes in the tumour microenvironments, whereas 38 (40%) of the cases were negative for PD-L1	Frequent discordance of PD-L1 expression between primary and metastatic tumours would indicate that the tissue typing for PD-L1 expression should ideally be performed on the metastatic tumour if PD-L1 positivity is included as an eligibility criterion in anti-PD-1/PD-L1 clinical trials
55	Seiwert et al. 2016 [55]	81 out of 104 (84%) screened patients were positive for PD-L1 at the 1% cut-off level	PD-L1 expression by IHC was predictive of best overall response and improved progression-free survival. Greater anti-tumour activity was recorded in OSCC that expressed higher levels of PD-L1
56	Shayan et al. 2017 [56]	Expression of macrophage PD-L1 was increased by approximately one-fold change when incubated with cetuximab plus motolimod	Addition of a PD-1 inhibitor to the combination of cetuximab and motolimod would further augment the antitumor response. Otolimod plus cetuximab decreased induction of Treg and reduced markers of suppression, including CTLA-4
57	Singh et al. 2017 [57]	PD-L1 was significantly up-regulated from 1 to 2 fold changes in most of the tumours but downregulated in two tumours	Expression of PD-L1 was upregulated in 10 of the 12 studied tumours suggesting these subsets of tumours may be susceptible for checkpoint blockade therapy
58	Sridharan et al. 2016 [58]	All checkpoint receptor bearing cell populations increased following treatment. Low levels of soluble PD-L1 in all patients was found	Radiation-induced effects on the local tumour microenvironment in OSCC patients may translate into quantifiable immune effects in circulating immune mediators, T cell receptor repertoires, and potential anti-tumour antibody responses. Higher baseline levels of soluble PD-L1 correlated with nodal status, higher levels in patients with node-positive disease
59	Strati et al. 2017 [59]	Median fold change of PD-L1 expression in the EpCAM- PBMC fraction was 1.03 in controls and 0.39 in OSCC samples , and in the EpCAM+ PBMC fraction was 1.28 in controls and 2.70 in OSCC samples	PD-L1 expression in the EpCAM+ CTC fraction may evolve during treatment and this modulation may inform clinical trial design of the sequence of chemotherapy and/or radiation with immunotherapy
60	Straub et al. 2016 [60]	PD-L1 expression in at least 5% of tumour cells was found in 36/80 of OSCCs. 41/79 cases contained PD-1 positive TILs. In the SCC cohort with PD-L1 immuno-positive tumours, the risk of tumour related death was significantly increased	PD-L1 gene is amplified in OSCC and is accompanied by immunohistochemical PD-L1 protein over-expression. PD-L1 positive cases have a significantly higher risk for nodal

			metastasis at diagnosis and is associated with higher risk for overall tumour-related death and recurrence
61	Strauss et al. 2017 [61]	CD25 high cells had significantly increased expression of CTLA-4 compared to CD25 low cells (mean fluorescence intensity of 120 vs 15). Rapamycin enhanced CTLA-4 expression on CD35 high clones with mean fluorescence intensity increasing from 120 pre-treatment to 235 post treatment	CD62L, CTLA-4 and Foxp3 are the key molecules associated with high levels of suppression mediated by human Tregs
62	Takahashi et al. 2019 [62]	31 patients had OSCC with low PD-L1 expression, and 46 had high PD-L1 expression	There was no significant relationship between any form of T-cell infiltration and PD-L1 expression in tumour cells
63	Takakura et al. 2017 [63]	PD-L1 expression analysis in cancer nest specimens showed in the nNAC group, 8/10 specimens showed intermediate-to-strong expression of PD-L1 protein whereas 6/8 NAC group specimens showed weak expression	no association was found between the groups of NAC and nNAC patients and the disease-free survival
64	Troeltzsch et al. 2017 [64]	Marked PD-L1 expression was observed in 26/88 (29%) OSCC specimens and 73/88 (83%) showed considerable presence of PD-1–positive TILs. Relevant PD-L1 expression was noted significantly more often in cancers from mandibular and oral tongue than maxilla or soft palate. OSCC specimens with high PD-L1 expression displayed infiltration with PD-1+ TILs	PD-L1 expression in OSCC was associated with an increased metastasis and greater levels of infiltrating PD-1+ TILs. PD-L1 expression in OSCC might differ depending on its anatomic origin
65	Wang et al. 2019 [65]	Nimotuzumab therapy significantly increased the expression of TIM-3, LAG-3, IDO, PD-L1, and CTLA-4 in the tumour microenvironment of OSCC patients compared with baseline	Aberrant expressed LAG-3 and PD-L1 were more likely to be associated with a worse survival (hazard ratio = 1.504) and could be considered as indicators of poor prognosis in OSCC
66	Weber er al. 2018 [66]	PD-L1_4 and PD-L1_2 showed significantly increased expression, up to 3-fold, in OSCC compared to normal oral mucosa	PD-L1 expression in peripheral blood might be an indicator of the existence of metastatic disease via nodal involvement in OSCC. There is also an association between a systemic state of immune tolerance and a more aggressive tumour
67	Wirsing et al. 2018 [67]	PD-L1 expression in OSCC cells correlates with increased infiltration of CD4+ cells and small tumour size	PD-L1-expressing tumour cells correlated positively to a tumour microenvironment rich in CD4+ cells but had no prognostic significance. PD-1/PD-L1-targeted immunotherapy might be successful in tumours rich in CD4+ cells
68	Wong et al. 2006 [68]	No significant difference between samples and controls for all CTLA-4 phenotypes (Phenotype A frequency: 59.9% in OSCC vs	CTLA-4 A/A genotype polymorphism is associated with younger age of onset and poorer survival in OSCC patients

		60.5% in controls, and phenotype G frequency: 89.9% in OSCC vs 83.0% in controls)	
69	Wu et al. 2017 [69]	IHC staining showed that CTLA-4 was highly expressed on the TILs in the tumour microenvironment. PD-L1 and CTLA-4 were overexpressed in OSCC and VISTA was positively correlated with PD-L1 (r=0.342) and CTLA-4 (0.294)	VISTA is overexpressed in OSCC and is correlated with PD-L1 and CTLA-4 indicating that it may regulate antitumor immunity
70	Wu et al. 2019 [70]	PD-1 was co-expressed with TIGIT on human CD4+ and CD8+ T cells and TILs, and that this co-expression was higher on TILs than PBMCs (62.61% total cells vs 9.22% total cells)	The data indicated that blocking PD-1 and TIGIT corporately may elicit better antitumor effects
71	Xu et al. 2019 [71]	PD-L1 immunopositivity was more frequent in the salivary duct carcinoma ex-pleiomorphic adenoma group than in the salivary duct carcinoma de-novo group. PD-1 expression in immune cells was seen in 35 salivary duct carcinomas. PD-L1 and PD-1 expression did not predict the risk of lymph node metastases	Expression of tumour-specific antigens and over-expression of PD-1 and PD-L1 indicate that salivary duct carcinoma patients may benefit from novel immune therapy approaches
72	Yagyuu et al. 2017 [72]	Oral pre-cancerous lesion patients with subepithelial PD-L1+ cell count below median showed a 5-year malignant-free survival rate of 96.8%, whereas the survival rate for those with above median counts showed a decreased survival rate of 86.1%. For every additional increase in the number of subepithelial PD-L1+ cells and PD-L1 positivity score of epithelium, the risk of malignant transformation increased 1.07 and 3.38 times, respectively	Subepithelial PD-L1-positive cell count and epithelial PD-L1 positivity were significantly associated with malignant transformation, whereas low subepithelial PD-L1 expression was associated with greater 5-year malignant free survival
73	Yang et al. 2019 [73]	Plasmacytoid dendritic cell infiltration was positively correlated with PD-1 (r = 0.3628), LAG3 (r = 0.3241), and TIM3 (r = 0.4834) staining	Plasmacytoid dendritic cell high infiltration correlated with an adverse outcome in human primary OSCC patients
74	Yoo et al. 2019 [74]	PD-L1 expression was positive in 65.2% and negative in 34.8% of samples	Loss of MHC class I expression is significantly associated with a worse prognosis in PD-L1-positive OSCC (hazard ratio = 4.24) but had no significance in the PD-L1 negative group. The combination of MHC class I and PD-L1 might be useful to predict the clinical course of the disease
75	Youngnak-Piboonratanakit et al. 2004 [75]	PD-1 was expressed abundantly on sub-epithelial infiltrating cells (38% cells/mm^2) in all OLP cases. PD-L1 was also moderately expressed in sub-epithelial infiltrates (25% cells/mm^2) in all OLP cases	PD-1 and its ligands are expressed on infiltrating lymphocytes in OLP lesions

76	Zhou et al. 2012 [76]	Expression of PD-1 and PD-L1 on T cells in OLP patients was significantly higher than controls (PD-1: 16.16% vs 10.15%. PD-L1: 22.73% vs 12.41%). Serum sPD-1 concentration had no difference between patients with OLP and healthy subjects	The PD-1/PD-L1 pathway may play an important role in negatively regulating T cell-mediated immune response in OLP. PD-L1 levels on peripheral blood T cells may be a marker in monitoring the disease severity of OLP. Agonists targeting this pathway could be a therapeutic strategy for OLP

Abbreviations: oral cavity squamous cell carcinoma (OSCC), oral lichen planus (OLP), immunohistochemistry (IHC), oral leukoplakia (OLK), tumour infiltrating lymphocyte (TIL), peripheral blood mononuclear cell (PBMC), neoadjuvant chemotherapy (NAC), non-neoadjuvant chemotherapy (nNAC), V-domain Ig suppressor of T cell activation (VISTA), T-cell immunoglobulin and ITIM domain (TIGIT).

References

1. Aggarwal, S.; Sharma, S.C.; Das, S.N. Dynamics of regulatory T cells (Tregs) in patients with oral squamous cell carcinoma. *J. Surg. Oncol.* **2017**, *116*, 1103–1113
2. Ahn, H.; Yang, J.M.; Kim, H.; Chung, J.H.; Ahn, S.H.; Jeong, W.J.; Paik, J.H. Clinicopathologic implications of the miR-197/PD-L1 axis in oral squamous cell carcinoma. *Oncotarget* **2017**, *8*, 66178–66194.
3. Balermpas, P.; Rodel, F.; Krause, M.; Linge, A.; Lohaus, F.; Baumann, M.; Tinhofer, I.; Budach, V.; Sak, A.; Stuschke, M.; et al. The PD-1/PD-L1 axis and human papilloma virus in patients with head and neck cancer after adjuvant chemoradiotherapy: A multicentre study of the German Cancer Consortium Radiation Oncology Group (DKTK-ROG). *Int. J. Cancer* **2017**, *141*, 594–603.
4. Bauml, J.; Seiwert, T.Y.; Pfister, D.G.; Worden, F.; Liu, S.V.; Gilbert, J.; Saba, N.F.; Weiss, J.; Wirth, L.; Sukari, A.; et al. Pembrolizumab for Platinum- and Cetuximab-Refractory Head and Neck Cancer: Results From a Single-Arm, Phase II Study. *J. Clin. Oncol.* **2017**, *35*, 1542–1549.
5. Bharti, V.; Mohanti, B.K.; Das, S.N. Functional genetic variants of CTLA-4 and risk of tobacco-related oral carcinoma in high-risk North Indian population. *Hum. Immunol.* **2013**, *74*, 348–352.
6. Bhosale, P.G.; Cristea, S.; Ambatipudi, S.; Desai, R.S.; Kumar, R.; Patil, A.; Kane, S.; Borges, A.M.; Schaffer, A.A.; Beerenwinkel, N.; et al. Chromosomal Alterations and Gene Expression Changes Associated with the Progression of Leukoplakia to Advanced Gingivobuccal Cancer. *Transl. Oncol.* **2017**, *10*, 396–409.
7. Cai, Y.; Wang, F.; Liu, Q.; Li, Z.; Li, D.; Sun, Z. A novel humanized anti-PD-1 monoclonal antibody potentiates therapy in oral squamous cell carcinoma. *Investig. New Drugs* **2019**, *37*, 799–809.
8. Chen, T.C.; Wu, C.T.; Wang, C.P.; Hsu, W.L.; Yang, T.L.; Lou, P.J.; Ko, J.Y.; Chang, Y.L. Associations among pretreatment tumor necrosis and the expression of HIF-1alpha and PD-L1 in advanced oral squamous cell carcinoma and the prognostic impact thereof. *Oral Oncol.* **2015**, *51*, 1004–1010.
9. Chen, S.-C.; Chang, P.M.-H.; Wang, H.-J.; Tai, S.-K.; Chu, P.-Y.; Yang, M.-H. PD-L1 expression is associated with p16(INK4A) expression in non-oropharyngeal head and neck squamous cell carcinoma. *Oncol. Lett.* **2018**, *15*, 2259–2265.
10. Chen, X.J.; Tan, Y.Q.; Zhang, N.; He, M.J.; Zhou, G. Expression of programmed cell death-ligand 1 in oral squamous cell carcinoma and oral leukoplakia is associated with disease progress and CD8+ tumor-infiltrating lymphocytes. *Pathol. Res. Pract.* **2019**, *215*, 152418.
11. Cho, Y.A.; Yoon, H.J.; Lee, J.I.; Hong, S.P.; Hong, S.D. Relationship between the expressions of PD-L1 and tumor-infiltrating lymphocytes in oral squamous cell carcinoma. *Oral Oncol.* **2011**, *47*, 1148–1153.
12. Dong, J.; Cheng, L.; Zhao, M.; Pan, X.; Feng, Z.; Wang, D. Tim-3-expressing macrophages are functionally suppressed and expanded in oral squamous cell carcinoma due to virus-induced Gal-9 expression. *Tumour Biol.* **2017**, *39*,1010428317701651.

13. Du, G.H.; Qin, X.P.; Li, Q.; Zhou, Y.M.; Shen, X.M.; Tang, G.Y. The high expression level of programmed death-1 ligand 2 in oral lichen planus and the possible costimulatory effect on human T cells. *J. Oral Pathol. Med.* **2011**, *40*, 525–532.
14. Falco, A.; Angel, M.; Perez, J.F.; Cayol, F.; Pucella, I.; Gomez Abuin, G.; Florez, J.; Adelchanow, E.; Palazzo, J.; Palazzo, F.; et al. Immunotherapy in Advanced Head and Neck Squamous Cell Carcinoma: A Retrospective Evaluation in Latin America. *Biomed. J. Sci. Tech. Res.* **2019**, *17*, 12708–12712.
15. Fayette, J.; Guigay, J.; Le Tourneau, C.; Degardin, M.; Peyrade, F.; Neidhardt, E.M.; Sablin, M.P.; Even, C.; Orlandini, F.; Juzyna, B.; et al. Cabazitaxel in recurrent/metastatic squamous cell carcinoma of the head and neck: Phase II UNICANCER trial ORL03. *Oncotarget* **2017**, *8*, 51830–51839.
16. Feldman, R.; Gatalica, Z.; Knezetic, J.; Reddy, S.; Nathan, C.A.; Javadi, N.; Teknos, T. Molecular profiling of head and neck squamous cell carcinoma. *Head Neck* **2016**, *38* (Suppl. 1), E1625–E1638.
17. Ferris, R.L.; Blumenschein, G., Jr., Fayette, J.; Guigay, J.; Colevas, A.D.; Licitra, L.; Harrington, K.; Kasper, S.; Vokes, E.E.; Even, C.; et al. Nivolumab for Recurrent Squamous-Cell Carcinoma of the Head and Neck. *N. Engl. J. Med.* **2016**, *375*, 1856–1867.
18. Fiedler, M.; Weber, F.; Hautmann, M.G.; Haubner, F.; Reichert, T.E.; Klingelhoffer, C.; Schreml, S.; Meier, J.K.; Hartmann, A.; Ettl, T. Biological predictors of radiosensitivity in head and neck squamous cell carcinoma. *Clin. Oral Investig.* **2017**, *22*, 189–200.
19. Foy, J.P.; Bertolus, C.; Michallet, M.C.; Deneuve, S.; Incitti, R.; Bendriss-Vermare, N.; Albaret, M.A.; Ortiz-Cuaran, S.; Thomas, E.; Colombe, A.; et al. The immune microenvironment of HPV-negative oral squamous cell carcinoma from never-smokers and never-drinkers patients suggests higher clinical benefit of IDO1 and PD1/PD-L1 blockade. *Ann. Oncol.* **2017**, *28*, 1934–1941.
20. Gasparoto, T.H.; de Souza Malaspina, T.S.; Benevides, L.; de Melo, E.J., Jr.; Costa, M.R.; Damante, J.H.; Ikoma, M.R.; Garlet, G.P.; Cavassani, K.A.; da Silva, J.S.; et al. Patients with oral squamous cell carcinoma are characterized by increased frequency of suppressive regulatory T cells in the blood and tumor microenvironment. *Cancer Immunol. Immunother.* **2010**, *59*, 819–828.
21. Ghapanchi, J.; Ghaderi, H.; Haghshenas, M.R.; Jamshidi, S.; Rezazadeh, F.; Azad, A.; Farzin, M.; Derafshi, R.; Kalantari, A.H. Observational Molecular Case-Control Study of Genetic Polymorphisms 1 in Programmed Cell Death Protein-1 in Patients with Oral Lichen Planus. *Asian Pac. J. Cancer Prev.* **2019**, *20*, 421–424.
22. Goltz, D.; Gevensleben, H.; Dietrich, J.; Schroeck, F.; de Vos, L.; Droege, F.; Kristiansen, G.; Schroeck, A.; Landsberg, J.; Bootz, F.; et al. PDCD1 (PD-1) promoter methylation predicts outcome in head and neck squamous cell carcinoma patients. *Oncotarget* **2017**, *8*, 41011–41020.
23. Groeger, S.; Howaldt, H.P.; Raifer, H.; Gattenloehner, S.; Chakraborty, T.; Meyle, J. Oral Squamous Carcinoma Cells Express B7-H1 and B7-DC Receptors in Vivo. *Pathol. Oncol. Res.* **2017**, *23*, 99–110.
24. Hanna, G.J.; Woo, S.B.; Li, Y.Y.; Barletta, J.A.; Hammerman, P.S.; Lorch, J.H. Tumor PD-L1 expression is associated with improved survival and lower recurrence risk in young women with oral cavity squamous cell carcinoma. *Int. J. Oral Maxillofac. Surg.* **2017**, *47*, 568–577.
25. Hanna, G.J.; Liu, H.; Jones, R.E.; Bacay, A.F.; Lizotte, P.H.; Ivanova, E.V.; Bittinger, M.A.; Cavanaugh, M.E.; Rode, A.J.; Schoenfeld, J.D.; et al. Defining an inflamed tumor immunophenotype in recurrent, metastatic squamous cell carcinoma of the head and neck. *Oral Oncol.* **2017**, *67*, 61–69.
26. Jie, H.B.; Gildener-Leapman, N.; Li, J.; Srivastava, R.M.; Gibson, S.P.; Whiteside, T.L.; Ferris, R.L. Intratumoral regulatory T cells upregulate immunosuppressive molecules in head and neck cancer patients. *Br. J. Cancer* **2013**, *109*, 2629–2635.
27. Kammerer, P.W.; Toyoshima, T.; Schoder, F.; Kammerer, P.; Kuhr, K.; Brieger, J.; Al-Nawas, B. Association of T-cell regulatory gene polymorphisms with oral squamous cell carcinoma. *Oral Oncol.* **2010**, *46*, 543–548.
28. Katou, F.; Ohtani, H.; Watanabe, Y.; Nakayama, T.; Yoshie, O.; Hashimoto, K. Differing phenotypes between intraepithelial and stromal lymphocytes in early-stage tongue cancer. *Cancer Res.* **2007**, *67*, 11195–11201.

29. Larkins, E.; Blumenthal, G.M.; Yuan, W.; He, K.; Sridhara, R.; Subramaniam, S.; Zhao, H.; Liu, C.; Yu, J.; Goldberg, K.B.; et al. FDA Approval Summary: Pembrolizumab for the Treatment of Recurrent or Metastatic Head and Neck Squamous Cell Carcinoma with Disease Progression on or After Platinum-Containing Chemotherapy. *Oncolologist* **2017**, *22*, 873–878.
30. Lecerf, C.; Kamal, M.; Vacher, S.; Chemlali, W.; Schnitzler, A.; Morel, C.; Dubot, C.; Jeannot, E.; Meseure, D.; Klijanienko, J.; et al. Immune gene expression in head and neck squamous cell carcinoma patients. *Eur. J. Cancer* **2019**, *121*, 210–223.
31. Lechner, A.; Schlosser, H.; Rothschild, S.I.; Thelen, M.; Reuter, S.; Zentis, P.; Shimabukuro-Vornhagen, A.; Theurich, S.; Wennhold, K.; Garcia-Marquez, M.; et al. Characterization of tumor-associated T-lymphocyte subsets and immune checkpoint molecules in head and neck squamous cell carcinoma. *Oncotarget* **2017**, *8*, 44418–44433.
32. Leduc, C.; Adam, J.; Louvet, E.; Sourisseau, T.; Dorvault, N.; Bernard, M.; Maingot, E.; Faivre, L.; Cassin-Kuo, M.-S.; Boissier, E.; et al. TPF induction chemotherapy increases PD-L1 expression in tumour cells and immune cells in head and neck squamous cell carcinoma. *ESMO Open* **2018**, *3*, e000257.
33. Lin, Y.M.; Sung, W.W.; Hsieh, M.J.; Tsai, S.C.; Lai, H.W.; Yang, S.M.; Shen, K.H.; Chen, M.K.; Lee, H.; Yeh KT et al.: High PD-L1 Expression Correlates with Metastasis and Poor Prognosis in Oral Squamous Cell Carcinoma. *PLoS ONE* **2015**, *10*, e0142656.
34. Linedale, R.; Schmidt, C.; King, B.T.; Ganko, A.G.; Simpson, F.; Panizza, B.J.; Leggatt, G.R. Elevated frequencies of CD8 T cells expressing PD-1, CTLA-4 and Tim-3 within tumour from perineural squamous cell carcinoma patients. *PLoS ONE* **2017**, *12*, e0175755.
35. Malaspina, T.S.; Gasparoto, T.H.; Costa, M.R.; de Melo, E.F., Jr.; Ikoma, M.R.; Damante, J.H.; Cavassani, K.A.; Garlet, G.P.; da Silva, J.S.; Campanelli, A.P. Enhanced programmed death 1 (PD-1) and PD-1 ligand (PD-L1) expression in patients with actinic cheilitis and oral squamous cell carcinoma. *Cancer Immunol. Immunother.* **2011**, *60*, 965–974.
36. Malm, I.J.; Bruno, T.C.; Fu, J.; Zeng, Q.; Taube, J.M.; Westra, W.; Pardoll, D.; Drake, C.G.; Kim, Y.J. Expression profile and in vitro blockade of programmed death-1 in human papillomavirus-negative head and neck squamous cell carcinoma. *Head Neck* **2015**, *37*, 1088–1095.
37. Maruse, Y.; Kawano, S.; Jinno, T.; Matsubara, R.; Goto, Y.; Kaneko, N.; Sakamoto, T.; Hashiguchi, Y.; Moriyama, M.; Toyoshima, T.; et al. Significant association of increased PD-L1 and PD-1 expression with nodal metastasis and a poor prognosis in oral squamous cell carcinoma. *Int. J. Oral Maxillofac. Surg.* **2018**, *47*, 836–845.
38. Mattox, A.; Lee, J.; Westra, W.H.; Pierce, R.H.; Ghossein, R.; Faquin, W.C.; Diefenbach, T.J.; Morris, L.G.; Lin, D.T.; Wirth, L.J.; et al. PD-1 expression in head and neck squamous cell carcinomas derives primarily from functionally anergic CD4+ TILs in the presence of PD-L1+ TAMs. *Cancer Res.* **2017**, *77*, 6365–6374.
39. Moratin, J.; Metzger, K.; Safaltin, A.; Herpel, E.; Hoffmann, J.; Freier, K.; Hess, J.; Horn, D. Upregulation of PD-L1 and PD-L2 in neck node metastases of head and neck squamous cell carcinoma. *Head Neck* **2019**, *41*, 2484–2491.
40. Moreira, G.; Fulgencio, L.B.; Ef, D.E.M.; Leles, C.R.; Batista, A.C.; Ta, D.A.S. T regulatory cell markers in oral squamous cell carcinoma: Relationship with survival and tumor aggressiveness. *Oncol. Lett.* **2010**, *1*, 127–132.
41. Muller, T.; Braun, M.; Dietrich, D.; Aktekin, S.; Hoft, S.; Kristiansen, G.; Goke, F.; Schrock, A.; Bragelmann, J.; Held, S.A.E.; et al. PD-L1: A novel prognostic biomarker in head and neck squamous cell carcinoma. *Oncotarget* **2017**, *8*, 52889–52900.
42. Naruse, T.; Yanamoto, S.; Okuyama, K.; Ohmori, K.; Tsuchihashi, H.; Furukawa, K.; Yamada, S.I.; Umeda, M. Immunohistochemical Study of PD-1/PD-L1 Axis Expression in Oral Tongue Squamous Cell Carcinomas: Effect of Neoadjuvant Chemotherapy on Local Recurrence. *Pathol. Oncol. Res.* **2019**, *26*, 735–742.
43. Ngamphaiboon, N.; Chureemas, T.; Siripoon, T.; Arsa, L.; Trachu, N.; Jiarpinitnun, C.; Pattaranutaporn, P.; Sirachainan, E.; Larbcharoensub, N. Characteristics and impact of programmed death-ligand 1 expression, CD8+ tumor-infiltrating lymphocytes, and p16 status in head and neck squamous cell carcinoma. *Med. Oncol.* **2019**, *36*, 21.
44. Okada, S.; Itoh, K.; Ishihara, S.; Shimada, J.; Kato, D.; Tsunezuka, H.; Miyata, N.; Hirano, S.; Teramukai, S.; Inoue, M. Significance of PD-L1 expression in pulmonary metastases from head and neck squamous cell carcinoma. *Surg. Oncol.* **2018**, *27*, 259–265.

45. Oliveira-Costa, J.P.; de Carvalho, A.F.; da Silveira da, G.G.; Amaya, P.; Wu, Y.; Park, K.J.; Gigliola, M.P.; Lustberg, M.; Buim, M.E.; Ferreira EN et al.: Gene expression patterns through oral squamous cell carcinoma development: PD-L1 expression in primary tumor and circulating tumor cells. *Oncotarget* **2015**, *6*, 20902–20920.
46. Pekiner, F.N.; Demirel, G.Y.; Borahan, M.O.; Ozbayrak, S. Evaluation of cytotoxic T-cell activation, chemokine receptors, and adhesion molecules in blood and serum in patients with oral lichen planus. *J. Oral Pathol. Med.* **2012**, *41*, 484–489.
47. Poropatich, K.; Fontanarosa, J.; Swaminathan, S.; Dittmann, D.; Chen, S.; Samant, S.; Zhang, B. Comprehensive T-cell immunophenotyping and next-generation sequencing of human papillomavirus (HPV)-positive and HPV-negative head and neck squamous cell carcinomas. *J. Pathol.* **2017**, *243*, 354–365.
48. Quan, H.; Fang, L.; Pan, H.; Deng, Z.; Gao, S.; Liu, O.; Wang, Y.; Hu, Y.; Fang, X.; Yao, Z.; et al. An adaptive immune response driven by mature, antigen-experienced T and B cells within the microenvironment of oral squamous cell carcinoma. *Int. J. Cancer* **2016**, *138*, 2952–2962.
49. Rasmussen, J.H.; Lelkaitis, G.; Hakansson, K.; Vogelius, I.R.; Johannesen, H.H.; Fischer, B.M.; Bentzen, S.M.; Specht, L.; Kristensen, C.A.; von Buchwald, C.; et al. Intratumor heterogeneity of PD-L1 expression in head and neck squamous cell carcinoma. *Br. J. Cancer* **2019**, *120*, 1003–1006.
50. Ryu, H.J.; Kim, E.K.; Heo, S.J.; Cho, B.C.; Kim, H.R.; Yoon, S.O. Architectural patterns of p16 immunohistochemical expression associated with cancer immunity and prognosis of head and neck squamous cell carcinoma. *Acta Pathol. Microbiol. Et Immunol. Scand.* **2017**, *125*, 974–984.
51. Saada-Bouzid, E.; Defaucheux, C.; Karabajakian, A.; Coloma, V.P.; Servois, V.; Paoletti, X.; Even, C.; Fayette, J.; Guigay, J.; Loirat, D.; et al. Hyperprogression during anti-PD-1/PD-L1 therapy in patients with recurrent and/or metastatic head and neck squamous cell carcinoma. *Ann. Oncol.* **2017**, *28*, 1605–1611.
52. Sablin, M.P.; Dubot, C.; Klijanienko, J.; Vacher, S.; Ouafi, L.; Chemlali, W.; Caly, M.; Sastre-Garau, X.; Lappartient, E.; Mariani, O.; et al. Identification of new candidate therapeutic target genes in head and neck squamous cell carcinomas. *Oncotarget* **2016**, *7*, 47418–47430.
53. Satgunaseelan, L.; Gupta, R.; Madore, J.; Chia, N.; Lum, T.; Palme, C.E.; Boyer, M.; Scolyer, R.A.; Clark, J.R. Programmed cell death-ligand 1 expression in oral squamous cell carcinoma is associated with an inflammatory phenotype. *Pathology* **2016**, *48*, 574–580.
54. Scognamiglio, T.; Chen, Y.T. Beyond the Percentages of PD-L1-Positive Tumor Cells: Induced Versus Constitutive PD-L1 Expression in Primary and Metastatic Head and Neck Squamous Cell Carcinoma. *Head Neck Pathol.* **2017**, *12*, 221–229.
55. Seiwert, T.Y.; Burtness, B.; Mehra, R.; Weiss, J.; Berger, R.; Eder, J.P.; Heath, K.; McClanahan, T.; Lunceford, J.; Gause, C.; et al. Safety and clinical activity of pembrolizumab for treatment of recurrent or metastatic squamous cell carcinoma of the head and neck (KEYNOTE-012): An open-label, multicentre, phase 1b trial. *Lancet Oncol.* **2016**, *17*, 956–965.
56. Shayan, G.; Kansy, B.A.; Gibson, S.P.; Srivastava, R.M.; Bryan, J.K.; Bauman, J.E.; Ohr, J.; Kim, S.; Duvvuri, U.; Clump, D.A.; et al. Phase Ib Study of Immune Biomarker Modulation with Neoadjuvant Cetuximab and TLR8 Stimulation in Head and Neck Cancer to Overcome Suppressive Myeloid Signals. *Clin. Cancer Res.* **2018**, *24*, 62–72.
57. Singh, R.; De Sarkar, N.; Sarkar, S.; Roy, R.; Chattopadhyay, E.; Ray, A.; Biswas, N.K.; Maitra, A.; Roy, B. Analysis of the whole transcriptome from gingivo-buccal squamous cell carcinoma reveals deregulated immune landscape and suggests targets for immunotherapy. *PLoS ONE* **2017**, *12*, e0183606.
58. Sridharan, V.; Margalit, D.N.; Lynch, S.A.; Severgnini, M.; Zhou, J.; Chau, N.G.; Rabinowits, G.; Lorch, J.H.; Hammerman, P.S.; Hodi, F.S.; et al. Definitive chemoradiation alters the immunologic landscape and immune checkpoints in head and neck cancer. *Br. J. Cancer* **2016**, *115*, 252–260.
59. Strati, A.; Koutsodontis, G.; Papaxoinis, G.; Angelidis, I.; Zavridou, M.; Economopoulou, P.; Kotsantis, I.; Avgeris, M.; Mazel, M.; Perisanidis, C.; et al. Prognostic significance of PD-L1 expression on circulating tumor cells in patients with head and neck squamous cell carcinoma. *Ann. Oncol.* **2017**, *28*, 1923–1933.
60. Straub, M.; Drecoll, E.; Pfarr, N.; Weichert, W.; Langer, R.; Hapfelmeier, A.; Gotz, C.; Wolff, K.D.; Kolk, A.; Specht, K. CD274/PD-L1 gene amplification and PD-L1 protein expression are common events in squamous cell carcinoma of the oral cavity. *Oncotarget* **2016**, *7*, 12024–12034.
61. Strauss, L.; Bergmann, C.; Whiteside, T.L. Functional and phenotypic characteristics of CD4+CD25highFoxp3+ Treg clones obtained from peripheral blood of patients with cancer. *Int. J. Cancer* **2007**, *121*, 2473–2483.

62. Takahashi, H.; Sakakura, K.; Arisaka, Y.; Tokue, A.; Kaira, K.; Tada, H.; Higuchi, T.; Okamoto, A.; Tsushima, Y.; Chikamatsu, K. Clinical and Biological Significance of PD-L1 Expression Within the Tumor Microenvironment of Oral Squamous Cell Carcinoma. *Anticancer Res.* **2019**, *39*, 3039–3046.
63. Takakura, H.; Domae, S.; Ono, T.; Sasaki, A. The Immunological Impact of Chemotherapy on the Tumor Microenvironment of Oral Squamous Cell Carcinoma. *Acta Med. Okayama* **2017**, *71*, 219–226.
64. Troeltzsch, M.; Woodlock, T.; Pianka, A.; Otto, S.; Troeltzsch, M.; Ehrenfeld, M.; Knosel, T. Is There Evidence for the Presence and Relevance of the PD-1/PD-L1 Pathway in Oral Squamous Cell Carcinoma? Hints From an Immunohistochemical Study. *J. Oral Maxillofac. Surg.* **2017**, *75*, 969–977.
65. Wang, H.; Mao, L.; Zhang, T.; Zhang, L.; Wu, Y.; Guo, W.; Hu, J.; Ju, H.; Ren, G. Altered expression of TIM-3, LAG-3, IDO, PD-L1, and CTLA-4 during nimotuzumab therapy correlates with responses and prognosis of oral squamous cell carcinoma patients. *J. Oral Pathol. Med.* **2019**, *48*, 669–676.
66. Weber, M.; Wehrhan, F.; Baran, C.; Agaimy, A.; Büttner-Herold, M.; Preidl, R.; Neukam, F.W.; Ries, J. PD-L1 expression in tumor tissue and peripheral blood of patients with oral squamous cell carcinoma. *Oncotarget* **2017**, *8*, 112584–112597.
67. Wirsing, A.M.; Ervik, I.K.; Seppola, M.; Uhlin-Hansen, L.; Steigen, S.E.; Hadler-Olsen, E. Presence of high-endothelial venules correlates with a favorable immune microenvironment in oral squamous cell carcinoma. *Mod. Pathol.* **2018**, *31*, 910–922.
68. Wong, Y.K.; Chang, K.W.; Cheng, C.Y.; Liu, C.J. Association of CTLA-4 gene polymorphism with oral squamous cell carcinoma. *J. Oral Pathol. Med.* **2006**, *35*, 51–54.
69. Wu, L.; Deng, W.W.; Huang, C.F.; Bu, L.L.; Yu, G.T.; Mao, L.; Zhang, W.F.; Liu, B.; Sun, Z.J. Expression of VISTA correlated with immunosuppression and synergized with CD8 to predict survival in human oral squamous cell carcinoma. *Cancer Immunol. Immunother.* **2017**, *66*, 627–636.
70. Wu, L.; Mao, L.; Liu, J.F.; Chen, L.; Yu, G.T.; Yang, L.L.; Wu, H.; Bu, L.L.; Kulkarni, A.B.; Zhang, W.F.; et al. Blockade of TIGIT/CD155 Signaling Reverses T-cell Exhaustion and Enhances Antitumor Capability in Head and Neck Squamous Cell Carcinoma. *Cancer Immunol. Res.* **2019**, *7*, 1700–1713.
71. Xu, B.; Jungbluth, A.A.; Frosina, D.; Alzumaili, B.; Aleynick, N.; Slodkowska, E.; Higgins, K.; Ho, A.; Morris, L.; Ghossein, R.; et al. The immune microenvironment and expression of PD-L1, PD-1, PRAME and MHC I in salivary duct carcinoma. *Histopathology* **2019**, *75*, 672–682.
72. Yagyuu, T.; Hatakeyama, K.; Imada, M.; Kurihara, M.; Matsusue, Y.; Yamamoto, K.; Obayashi, C.; Kirita, T. Programmed death ligand 1 (PD-L1) expression and tumor microenvironment: Implications for patients with oral precancerous lesions. *Oral Oncol.* **2017**, *68*, 36–43.
73. Yang, L.L.; Mao, L.; Wu, H.; Chen, L.; Deng, W.W.; Xiao, Y.; Li, H.; Zhang, L.; Sun, Z.J. pDC depletion induced by CD317 blockade drives the antitumor immune response in head and neck squamous cell carcinoma. *Oral Oncol.* **2019**, *96*, 131–139.
74. Yoo, S.H.; Keam, B.; Ock, C.Y.; Kim, S.; Han, B.; Kim, J.W.; Lee, K.W.; Jeon, Y.K.; Jung, K.C.; Chung EJ et al.: Prognostic value of the association between MHC class I downregulation and PD-L1 upregulation in head and neck squamous cell carcinoma patients. *Sci. Rep.* **2019**, *9*, 7680.
75. Youngnak-Piboonratanakit, P.; Tsushima, F.; Otsuki, N.; Igarashi, H.; Machida, U.; Iwai, H.; Takahashi, Y.; Omura, K.; Yokozeki, H.; Azuma, M. The expression of B7-H1 on keratinocytes in chronic inflammatory mucocutaneous disease and its regulatory role. *Immunol. Lett.* **2004**, *94*, 215–222.
76. Zhou, G.; Zhang, J.; Ren, X.W.; Hu, J.Y.; Du, G.F.; Xu, X.Y. Increased B7-H1 expression on peripheral blood T cells in oral lichen planus correlated with disease severity. *J. Clin. Immunol.* **2012**, *32*, 794–801.
